# Dual Biomarker Strategies for Liquid Biopsy: Integrating Circulating Tumor Cells and Circulating Tumor DNA for Enhanced Tumor Monitoring

**DOI:** 10.3390/bios15020074

**Published:** 2025-01-28

**Authors:** Ga Young Moon, Basak Dalkiran, Hyun Sung Park, Dongjun Shin, Chaeyeon Son, Jung Hyun Choi, Seha Bang, Hosu Lee, Il Doh, Dong Hyung Kim, Woo-jin Jeong, Jiyoon Bu

**Affiliations:** 1 Department of Biological Sciences and Bioengineering, Inha University, 100 Inha-ro, Michuhol-gu, Incheon 22212, Republic of Korea; 22241217@inha.edu (G.Y.M.); basakdalkiran@inha.edu (B.D.); hyunseong@inha.edu (H.S.P.); sdj8876@inha.edu (D.S.); chaeyeon1531@inha.edu (C.S.); hermes112@inha.edu (J.H.C.); jesus990318@inha.edu (S.B.); 12200945@inha.edu (H.L.); 2 Division of Biomedical Metrology, Korea Research Institute of Standards and Science, 267 Gajeongno, Yuseong-gu, Daejeon 34113, Republic of Korea; il.doh@kriss.re.kr (I.D.); donghyung.kim@kriss.re.kr (D.H.K.); 3 Department of Biological Engineering, Inha University, 100 Inha-ro, Michuhol-gu, Incheon 22212, Republic of Korea; 4 Biohybrid Systems Research Center, Inha University, 100 Inha-ro, Michuhol-gu, Incheon 22212, Republic of Korea

**Keywords:** liquid biopsy, circulating tumor DNA (ctDNA), circulating tumor cells (CTCs), diagnostic biomarker, tumor diagnosis

## Abstract

The liquid biopsy has gained significant attention in cancer diagnostics, with circulating tumor cells (CTCs) and circulating tumor DNA (ctDNA) being recognized as key biomarkers for tumor detection and monitoring. However, each biomarker possesses inherent limitations that restrict its standalone clinical utility, such as the rarity and heterogeneity of CTCs and the variable sensitivity and specificity of ctDNA assays. This highlights the necessity of integrating both biomarkers to maximize diagnostic and prognostic potential, offering a more comprehensive understanding of the tumor biology and therapeutic response. In this review, we summarize clinical studies that have explored the combined analysis of CTCs and ctDNA as biomarkers, providing insights into their synergistic value in diverse tumor types. Specifically, this paper examines the individual advantages and limitations of CTCs and ctDNA, details the findings of combined biomarker studies across various cancers, highlights the benefits of dual biomarker approaches over single-biomarker strategies, and discusses future prospects for advancing personalized oncology through liquid biopsies. By offering a comprehensive overview of clinical studies combining CTCs and ctDNA, this review serves as a guideline for researchers and clinicians aiming to enhance biomarker-based strategies in oncology and informs biosensor design for improved biomarker detection.

## 1. Introduction

Cancer remains one of the leading causes of mortality worldwide. While advancements in therapeutic strategies have improved survival outcomes in specific tumor cases, the prognosis for many cancers remains poor, particularly when the tumor is diagnosed at advanced stages. Late-stage diagnoses significantly limit therapeutic options and reduce survival rates [1,2]. These challenges emphasize the necessity of early diagnosis, identifying the most effective treatment options, and continuously monitoring disease progression, which can be facilitated by strategies that capture the dynamic and heterogeneous nature of tumors [3].

The tissue biopsy is currently regarded as the gold standard for cancer diagnosis and molecular profiling. However, this approach is inherently limited by its static nature, offering only a single time-point snapshot of the tumor’s molecular features. Furthermore, the tissue biopsy relies on small specimens obtained from localized tumor sites, which might fail to capture the spatial heterogeneity of the tumor, as well as the temporal heterogeneity [4,5,6]. This heterogeneity, a hallmark of cancer, significantly influences therapeutic resistance and disease progression, making it difficult to fully characterize the complexity of the tumor biology. Additionally, the tissue biopsy is invasive, requiring surgical or needle-based procedures that can result in complications such as bleeding, infection, or damage to surrounding tissues. In certain cases, tissue biopsy may not be feasible (e.g., liver), particularly when tumors are located in anatomically challenging regions or when patient health conditions preclude invasive procedures. These limitations demonstrate the need for alternative diagnostic approaches capable of addressing the constraints associated with conventional tissue biopsy.

Alternatively, the liquid biopsy has gained significant clinical interest as a minimally invasive and dynamic approach for characterizing the tumor [7]. By analyzing circulating-tumor-derived components, such as circulating tumor cells (CTCs) and circulating tumor DNA (ctDNA) in blood samples, a liquid biopsy may overcome the limitations of a conventional tissue biopsy. Specifically, CTCs and ctDNA are released into the bloodstream from various regions, not only from the primary tumor but also from lymph nodes and metastatic sites. This enables a more comprehensive understanding of tumor heterogeneity, including both spatial and temporal variations. Additionally, since the liquid biopsy utilizes bodily fluids (e.g., blood) as a sample, it eliminates the need for invasive procedures, reducing the complications associated with tissue sampling [8,9,10,11]. Due to these advantages, the liquid biopsy also facilitates the real-time monitoring of tumor dynamics and enhances the ability to detect disease progression and treatment resistance. Moreover, the dynamic nature of the liquid biopsy supports continuous adjustments to therapeutic strategies based on the evolving molecular profile of the tumor, thereby enabling personalized cancer care.

**Figure 1:**
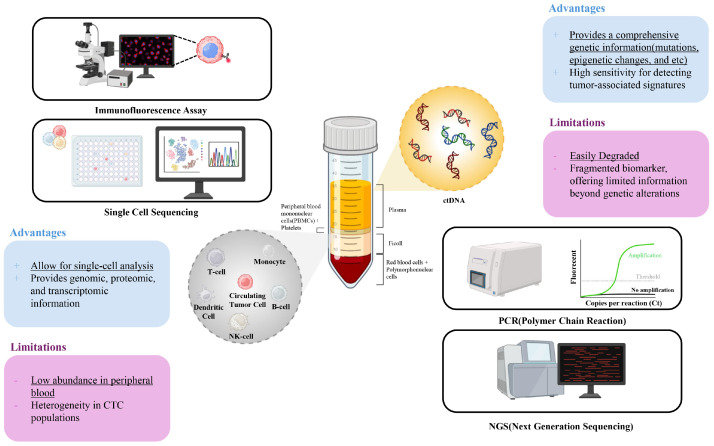
Advantages and limitations of circulating tumor cells (CTCs) and circulating tumor DNA (ctDNA) as biomarkers for tumor diagnosis and prognosis.

Among the various analytes applicable as tumor biomarkers for liquid biopsies, CTCs and ctDNA are the most extensively investigated and clinically significant biomarkers (Figure 1). CTCs are viable tumor cells that have detached from the primary or metastatic sites and entered the bloodstream, providing cellular information of the tumor [8]. In contrast, ctDNA comprises DNA fragments released into the circulation through tumor cell apoptosis or necrosis, reflecting the genetic and epigenetic alterations within the tumor [12]. Both biomarkers exhibit unique advantages, including their non-invasive nature, potential for real-time monitoring, and ability to provide the molecular characteristics of the tumor. Collectively, these biomarkers present an opportunity to enhance cancer diagnostics, prognostics, and therapeutic monitoring.

Despite their advantages, CTC- and ctDNA-based liquid biopsy approaches face distinct technical and biological challenges that limit their standalone clinical utility. CTCs, being rare cells shed into the bloodstream from tumors, are often present in extremely low numbers (0–100 cells per 1 mL blood), making their isolation and detection technically difficult [8]. The heterogeneity of CTCs further complicates their isolation, as well as characterization, as they can exhibit diverse phenotypes and genetic profiles depending on their origin and state of progression. Additionally, the viability of CTCs during collection and analysis is a critical factor that may affect the accuracy and reproducibility of downstream analyses [13]. On the other hand, ctDNA, which consists of fragmented DNA released from apoptotic and necrotic tumor cells, faces challenges related to its low abundance in the bloodstream, particularly in early-stage cancers [14]. The detection of ctDNA is further complicated by the presence of circulating cell-free DNA (cfDNA) from non-tumor sources, which can dilute tumor-specific signals. Moreover, ctDNA only reflects the genetic alterations present in the dying tumor cells at a specific timepoint, potentially missing active and viable tumor cell populations [12].

Given these challenges, combining CTCs and ctDNA provides a complementary approach that utilizes the advantages of both biomarkers while mitigating their individual limitations. This review focuses on clinical studies employing the integrated analysis of CTCs and ctDNA, emphasizing their synergistic potential to address the limitations of single-biomarker strategies. Furthermore, this review provides a comprehensive analysis of their advantages and applications across various tumor types and discusses future directions for advancing multimodal liquid biopsy platforms in cancer diagnosis and prognostic monitoring. This manuscript serves as a guideline for scientists and clinicians in the field of biosensors, particularly those interested in enhancing the clinical utility of their diagnostic devices.

## 2. Circulating Tumor Cells (CTCs): Clinical Utility and Limitations

Various technologies have been developed to isolate CTCs from blood samples, addressing the challenges associated with their rarity and heterogeneity. These include immunomagnetic separation, size-based filtration, and microfluidic devices. Each method utilizes the distinct properties of CTCs, such as their surface markers, size, or biophysical characteristics, to facilitate separation from other blood components. These advancements focus on enhancing the sensitivity and specificity of CTC isolation, which can ultimately enable more detailed downstream analyses for cancer diagnosis and prognostic monitoring.

Immunomagnetic separation is one of the most widely used methods for isolating CTCs, utilizing magnetic beads conjugated with antibodies that target specific surface markers, such as epithelial-cell adhesion molecule (EpCAM) [15,16]. The FDA-approved CellSearch system (Menarini Silicon Biosystems, Florence, Italy) is currently regarded as the gold standard for CTC isolation and detection. This assay uses immunomagnetic beads coated with anti-EpCAM antibodies (aEpCAM) to capture CTCs with an epithelial origin, which are subsequently identified based on EpCAM and/or cytokeratin positivity, nuclear staining, and CD45 negativity. However, reliance on the aEpCAM antibody as the primary capture agent presents significant limitations. CTCs that have undergone epithelial-to-mesenchymal transition (EMT) often downregulate EpCAM expression, leading to their exclusion during enrichment. Consequently, this method may fail to detect a substantial subset of clinically relevant CTCs, particularly those associated with metastatic progression [17].

To address this limitation, size-based filtration methods exploit the generally larger size of CTCs compared to other blood components, physically separating them through micro-sized filters [18]. This approach offers the advantage of rapid sample processing, making it suitable for high-throughput applications [19]. However, the heterogeneity of CTCs poses a significant limitation, as smaller CTCs—down to 4 µm in diameter—can pass through the filters undetected [20]. Additionally, the filter size directly impacts the trade-off between sensitivity and purity [21]. Reducing the filter size increases CTC capture efficiency but also results in greater contamination with non-CTC components, while larger filter sizes enhance purity but lead to the loss of smaller CTCs. These limitations highlight the need for optimization to balance sensitivity and specificity in size-based filtration approaches, a balance that is often difficult to achieve due to the heterogeneity of tumor cell sizes.

Microfluidic devices have also been employed for CTC isolation, utilizing label-free or size-based approaches to capture CTCs within a microfluidic chamber [22,23,24]. These systems offer high precision in fluid control and the ability to integrate multiple isolation mechanisms, such as immunocapture or size exclusion, to improve the capture efficiency. The advantages of microfluidic devices include reduced sample volumes and streamlined workflows, making them suitable in various clinical settings. However, their fabrication can be complex and costly, limiting scalability and broader implementation. Furthermore, the inherently low abundance of CTCs in the bloodstream presents a significant barrier to achieving clinically meaningful results, regardless of the isolation technique.

Enumerating and analyzing CTCs is also another critical step in their clinical application, with immunofluorescent staining remaining the gold standard for identifying isolated cells. This approach relies on markers, such as cytokeratin, EpCAM, CD45, and DAPI for CTC characterization. As aforementioned, the FDA-approved CellSearch system employs these criteria for CTC enumeration, but interpretation depends heavily on the clinician’s judgment, introducing variability in the results [25]. Furthermore, no universally accepted quantitative standard exists for defining CTC positivity, creating challenges in clinical decision-making. Emerging machine learning-based algorithms have been developed to automate CTC enumeration; however, these systems remain imperfect and are primarily used as adjuncts to assist clinicians rather than as standalone solutions.

To address the limitations of conventional CTC enumeration, advanced techniques, such as single-cell sequencing and biosensors have been developed [26]. These approaches provide detailed phenotypic and genomic information, offering insights into tumor biology that extend beyond enumeration. Despite their promise, these methods face persistent challenges, including low purity and insufficient CTC yields after isolation, which limit their clinical utility. Although CTCs provide valuable information on both the phenotypic and genomic characteristics of tumors, standalone CTC-based assays are constrained by these limitations. This highlights the necessity of integrating CTC analysis with complementary biomarkers, such as circulating tumor DNA, to enhance the reliability and applicability of liquid biopsy in clinical practice.

## 3. Circulating Tumor DNA (ctDNA): Introduction to Clinical Utility and Limitations 

Isolation and detection methods of cfDNA or ctDNA have been developed to enable the analysis of genetic material in a non-invasive manner. Various techniques have been employed for the isolation of cfDNA from plasma or serum samples. These methods include silica-based membrane extraction, magnetic bead-based extraction, and liquid-phase extraction using aqueous two-phase systems [27]. Silica-based membrane extraction utilizes the binding properties of silica to capture and purify cfDNA, while magnetic bead-based extraction relies on magnetic beads conjugated with DNA-binding molecules to isolate specific cfDNA fragments [28]. Liquid-phase extraction methods, on the other hand, separate cfDNA from other components in the sample based on differences in their partitioning behavior in an aqueous two-phase system [29]. These isolation methods aim to obtain high yields of cfDNA with minimal contamination from other cellular components.

ctDNA comprises only a small fraction of total circulating cell-free DNA (cfDNA), typically ranging from less than 0.1% to about 10% in plasma, depending on the tumor type and stage. Once cfDNA is isolated, ctDNA can be detected and quantified using various techniques [30]. Polymerase chain reaction (PCR) is commonly employed to amplify specific regions of cfDNA for detection and quantification. Real-time PCR, also known as quantitative PCR (qPCR), allows for the precise measurement of specific ctDNA levels by monitoring the amplification in real-time. NGS is another powerful tool for ctDNA analysis, enabling the comprehensive profiling of genetic alterations in ctDNA. NGS can provide information about the mutations, copy number variations, and other genomic alterations present in ctDNA. These detection methods allow for the analysis of ctDNA in a targeted or genome-wide manner, providing valuable insights into the genetic characteristics of tumors.

Despite advances in cfDNA isolation methods, several limitations remain. One major challenge is the low concentration of ctDNA in the blood, especially in early-stage cancers, which often leads to inadequate yields for downstream analysis [31]. Moreover, cfDNA is highly fragmented, with an average fragment size of 150–200 base pairs, making it difficult to separate tumor-derived ctDNA from background cfDNA originating from normal cells. This background cfDNA can dilute tumor-specific signals, reducing the sensitivity and specificity of the isolation process. Additionally, different isolation methods may yield varying quality and quantity of ctDNA, introducing variability that complicates standardization and reproducibility across laboratories. Factors, such as sample handling, storage conditions, and plasma preparation techniques, further affect cfDNA quality, creating potential obstacles to consistent clinical applications.

The detection of ctDNA also faces its own set of challenges, particularly in terms of sensitivity and specificity. PCR-based methods, while widely used, are limited in their ability to detect low-frequency mutations and provide only targeted information about specific genetic alterations [32]. Although NGS offers a more comprehensive view of ctDNA, it requires high-quality input DNA and remains costly and time-consuming, making it less accessible for routine clinical use. Moreover, NGS can generate false positives due to sequencing errors, particularly when detecting mutations at low allele frequencies, necessitating additional validation steps. Another limitation lies in the dynamic nature of ctDNA levels, which can fluctuate in response to treatment or disease progression, complicating the interpretation of results.

ctDNA also inherently lacks information on the physiological and phenotypic characteristics of tumors. This limitation poses challenges in clinical applications, particularly in guiding therapeutic decisions. For instance, ctDNA analysis may not provide comprehensive data on protein expression, cellular morphology, or tumor microenvironment interactions, all of which are critical factors in selecting optimal treatment strategies. Consequently, relying solely on ctDNA can lead to incomplete assessments, potentially affecting the efficacy of targeted therapies [33]. Moreover, the absence of phenotypic information in ctDNA analysis limits its ability to predict functional drug resistance mechanisms, which are often influenced by protein-level alterations and the cellular context. This gap demonstrates the importance of combining ctDNA analysis with other diagnostic approaches, such as CTC analysis, to obtain a more holistic view of tumor biology. By integrating multiple sources of information, clinicians can make more informed decisions regarding drug selection and treatment planning, thereby improving patient outcomes.

## 4. Integrated Analysis of CTCs and ctDNA

### 4.1. Advantages of Integrated Analysis of CTCs

Although CTC- and ctDNA-based liquid biopsy approaches individually provide variable clinical information about the pathological features of a tumor, their limitation indicates the need for an integrated analysis. CTCs, while offering phenotypic and morphological information about tumor cells, often suffer from low abundance in blood and technical challenges in isolation and enumeration. Conversely, ctDNA enables the highly sensitive and specific detection of genetic alterations but lacks information on the cellular context, including protein expression, cellular interactions, and phenotypic heterogeneity. Combining CTCs and ctDNA allows the advantages of each biomarker to compensate for the limitations of the other. For instance, the phenotypic insights from CTCs can complement the genetic data from ctDNA, providing a more holistic understanding of tumor dynamics [34]. This integration can improve the detection of metastasis, better predict drug-resistance mechanisms, and refine treatment stratification by combining cellular and genomic information.

The integration of CTC and ctDNA analyses is made possible by advancements in technology, particularly in NGS and machine learning algorithms [35,36,37,38]. NGS allows for the simultaneous profiling of ctDNA mutations and CTC-derived genomic material, providing a comprehensive molecular portrait of the tumor. Additionally, machine-learning-based algorithms have been employed to identify patterns in multi-omic datasets, enabling the correlation of ctDNA mutations with CTC phenotypes. Such integrated approaches not only enhance the diagnostic accuracy but may also enable the real-time monitoring of tumor evolution and therapeutic responses with high accuracy. These advancements also facilitate precision oncology by providing a deeper understanding of tumor heterogeneity and its clinical implications.

In the following sections, we introduce clinical studies that have employed CTCs and ctDNA together as biomarkers. To provide clarity, these studies are categorized based on tumor types, such as breast, lung, and gastrointestinal (GI) cancers, emphasizing the synergistic advantages of combining these biomarkers for specific cancer subtypes.

### 4.2. Breast Cancer

Breast cancer is a highly heterogeneous disease, exhibiting multiple subtypes that show significant variability in clinical behavior and responses to different therapeutic approaches [39,40,41]. This heterogeneity complicates the identification of biomarkers that can effectively capture the complexity of the disease, which is essential for enhancing diagnostic accuracy, guiding treatment strategies, and monitoring therapeutic efficacy. Established immunohistochemical markers, such as HER2, estrogen receptor (ER), and progesterone receptor (PR), have significantly contributed to the effective treatment of breast cancer [42,43,44,45]. However, these tissue-based biomarkers have inherent limitations in reflecting tumor evolution over time, as well as their spatial heterogeneity. Emerging liquid biopsy techniques, including ctDNA and CTCs, offer a minimally invasive strategy to address these limitations. While ctDNA provides highly sensitive genetic information, it lacks phenotypic context, and CTC detection is often constrained by low sensitivity in early-stage disease. The integration of CTCs and ctDNA has the potential to address these challenges by offering combined molecular and phenotypic information about tumors, facilitating a more detailed understanding of breast cancer.

**Figure 2:**
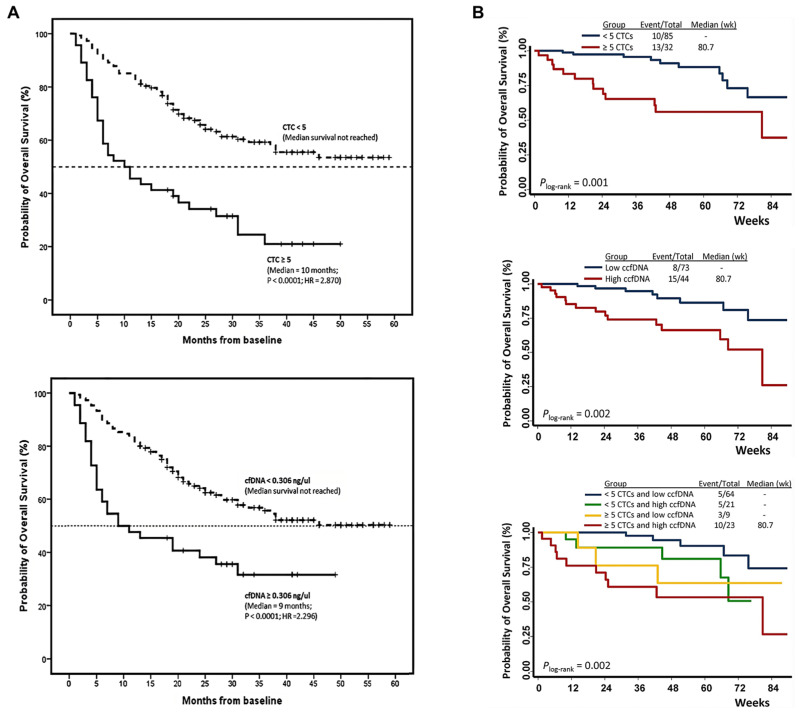
(**A**) Prognostic value of CTC counts and plasma cfDNA levels for estimating overall survival in metastatic breast cancer patients (reproduced under CC BY 4.0 DEED license, Copyright 2019, Springer Nature) [46]; (**B**) improved prognostic accuracy by combining CTC counts and cfDNA levels for estimating overall survival in metastatic breast cancer patients (reprinted from Ye, Zhong, et al. (2019), with permission from Elsevier) [47].

Fernandez-Garcia et al. quantitatively analyzed CTCs and cfDNA from the blood samples of 193 metastatic breast cancer (MBC) patients (Table 1) [46]. From this study, CTCs were enumerated using the CellSearch System, based on EpCAM-positive selection, with a threshold of ≥5 CTCs per 7.5 mL blood defining high counts, while cfDNA was quantitatively analyzed from 3 mL of plasma and quantified via qPCR. High cfDNA levels and CTC counts were strongly associated with poorer overall survival (OS), with hazard ratios of 2.296 and 2.870, respectively (*p* < 0.0001). When both markers were combined, the prognostic capability increased, with the median OS dropping from >59 months to 6 months. Specifically, the combined analysis demonstrated a sensitivity of 90% for predicting disease progression, significantly superior to 79% with cfDNA alone and 62% with CTCs alone (Figure 2A). The combined analysis of CTCs and cfDNA also outperformed conventional serum biomarkers used for the diagnosis of MBC, achieving an area under the curve (AUC) of 0.81 for overall survival, which was significantly higher than that of CA15-3 (AUC 0.65) and alkaline phosphatase (AP; AUC 0.62).

Similar findings have been reported from Ye et al. [47]. This study also enumerated CTCs using the CellSearch System, and cfDNA levels were quantitatively analyzed using qRT-PCR. Elevated CTCs (≥5 cells/7.5 mL) were associated with a 3.63-fold higher risk of death (*p* = 0.002), and high cfDNA levels were correlated with a 3.56-fold increased risk of death (*p* = 0.004) (Figure 2B). Notably, combining these biomarkers revealed a synergistic prognostic effect, with patients exhibiting high levels of both CTCs and cfDNA showing a 17.43-fold higher risk of death compared to those with low levels of both markers (*p* < 0.001). These findings emphasize the complementary clinical utility of CTCs and cfDNA for improving risk stratification and survival prediction in MBC patients.

The detection of specific tumor-associated mutations in ctDNA, when combined with CTC counts, can provide more detailed clinical information about the tumor. Radovich et al. analyzed the association of ctDNA and CTCs with disease recurrence in patients with triple-negative breast cancer (TNBC) upon neoadjuvant chemotherapy [48]. The trial enrolled 196 female patients with residual disease post-chemotherapy. ctDNA was sequenced using FoundationACT or FoundationOneLiquid assays, while CTC enumeration was conducted using an EpCAM-targeting microfluidic device. The detection of ctDNA was significantly associated with inferior distant-disease-free survival (DDFS), disease-free survival (DFS), and OS, as the DDFS probability at 24 months was 56% for ctDNA-positive patients versus 81% for ctDNA-negative patients. Meanwhile, CTC positivity alone showed an association with inferior outcomes, although the results were not as significant as ctDNA. However, combining ctDNA and CTCs improved the sensitivity for identifying disease recurrence (90% with both biomarkers compared to 79% with ctDNA alone and 62% with CTC alone). Furthermore, patients positive for both ctDNA and CTCs had the worst outcomes, with a median DDFS of 32.5 months compared to “not reached” for patients negative for both markers. The authors concluded that dual biomarker analysis can exhibit superior prognostic capability compared to single biomarkers alone, enabling better risk stratification and treatment guidance for TNBC patients.

In another study, mutation profiles were investigated using NGS for both CTCs and cfDNA obtained from the blood samples of MBC patients. Specifically, the authors identified tumor-associated mutations for CTCs and cfDNA across 50 cancer-related genes [34]. The authors found that significant mutational heterogeneity was observed among individual CTCs, even within the same patient sample. Meanwhile, cfDNA accurately reflected the mutations observed in individual CTCs and included additional mutations that were not detected in the primary tumor or some CTCs. As a result, the authors found that the elevated cfDNA levels and CTC counts (≥5 CTCs per 7.5 mL of blood) were significantly associated with poor overall survival. Furthermore, specific mutations (e.g., in PIK3CA, TP53, ESR1, and KRAS) detected in cfDNA and CTCs, provided further diagnostic capabilities for assessing the disease progression and therapeutic resistance.

Likewise, Keup et al. explored the genomic profiles of CTC-derived genomic DNA (gDNA) and cfDNA in HER2-negative MBC patients [49]. Using matched blood samples from 18 patients, targeted NGS was applied to assess genomic variants from CTC gDNA and cfDNA. Tumor-specific variants were found in 94% of patients when combining the two biomarkers, which was significantly higher than CTC (83%) or cfDNA (89%) alone. Specifically, 127 variants were identified in CTC gDNA, while 37 variants were detected in cfDNA; interestingly, only 28% of the variants overlapped between the two. Unique variants in CTC gDNA were more common in AR and ERBB2, whereas PIK3CA and ESR1 variants predominated in cfDNA, demonstrating the complementary nature of these biomarkers in tumor profiling. Notably, the ESR1 variants detected in both CTC gDNA and cfDNA were strongly correlated with survival outcomes (*p* < 0.05), with the combined analysis further enhancing prognostic capabilities. This study reveals that while either CTC gDNA or cfDNA alone may miss some clinically important information about their parental tumor cells, integrating both biomarkers can provide a more comprehensive molecular profile of the disease, significantly improving the analysis of tumor heterogeneity and guiding personalized treatment strategies.

**Table 1:** Combined analysis of circulating tumor cells and circulating tumor DNA for the diagnosis and molecular profiling of breast cancer.

Type	Samples	CTC		ctDNA		Summary	Ref.
		Isolation	Analysis	Extraction	Analysis		
MBC	193 MBC patients (cfDNA and CTC both analyzed)	CellSearch System (EpCAM-positive selection)	IF staining (EpCAM+, CK+, DAPI+, CD45−)	Qiagen Circulating Nucleic Acids Kit	Quantitatively analyzed using RealTime PCR System (96 bp single copy TaqMan assay)	High cfDNA levels and CTC counts associated with poor OS. Combined analysis of CTCs and cfDNA enhanced prognostic accuracy.	[46]
MBC	18 MBC patients with matched blood samples	AdnaTest EMT-2/StemCell Select System (targeting EpCAM, EGFR, and HER2)	IF staining (CK+, CD45−, DAPI+) Variants analyzed using customized QIAseq Targeted DNA Panel Kit	QIAamp Circulating Nucleic Acid Kit	Variants analyzed using customized QIAseq Targeted DNA Panel Kit (17 cancer-related genes)	94% of patients had tumor-specific variants when combining cfDNA and CTC gDNA. Unique variants observed in AR and ERBB2 for CTC gDNA and PIK3CA and ESR1 for cfDNA.	[49]
MBC	227 blood samples from 117 MBC patients	CellSearch System (EpCAM-positive selection)	IF staining (CK+, CD45−, DAPI+)	QIAamp Circulating Nucleic Acid Kit	Quantitatively analyzed using Qubit fluorometry and qPCR targeting ALU repeats	High CTC counts and cfDNA levels were independently associated with poor OS. Combined high levels showed >17-fold increased risk of death.	[47]
TNBC	196 TNBC patients (142 ctDNA; 123 CTC analyzed)	Anti-EpCAM magnetic beads with microfluidic device	IF staining (CK+, CD45−, DAPI+)	Qiagen Circulating Nucleic Acids Kit	FoundationACT and Foundation One Liquid assays (detection of point mutations, CNAs, and rearrangements)	Combination of ctDNA and CTCs associated with increased sensitivity for detecting DDFS and DFS. Patients positive for both markers had significantly worse outcomes.	[48]
MBC	57 MBC patients (57 CTC/ctDNA analyzed + 31 samples for targeted CTC sequencing)	DEPArray system (EpCAM-positive selection)	IF staining (EpCAM+, CK+, DAPI+, CD45−) NGS with AmpliSeq panel	QIAamp Circulating Nucleic Acid Kit	NGS with a AmpliSeq panel (50-gene panel) targeting mutations in PIK3CA, TP53, ESR1, KRAS, etc.	Significant mutational heterogeneity observed in CTCs. High cfDNA and CTC levels associated with poor overall survival.	[34]
BC	114 paired CTC and cfDNA samples including 79 BC patients	Anti-EpCAM magnetic bead-based positive selection	Methylation-specific PCR (MSP) targeting SOX17 promoter analyzed from DNA and KRT19 analyzed from mRNA using RTq-PCR	High Pure Viral nucleic acid kit	SOX17 promoter methylation analysis using MSP	SOX17 promoter methylation detected in 86.0% of primary tumors, with a strong correlation between cfDNA and CTC methylation patterns.	[50]

CTCs: circulating tumor cells; ctDNA: circulating tumor DNA; MBC: metastatic breast cancer; TNBC: triple-negative breast cancer; BC: breast cancer; IF staining: immunofluorescence staining; EpCAM: epithelial cell adhesion molecule; DEP: dielectrophoresis; CK: cytokeratin; PCR: polymerase chain reaction; qPCR: quantitative PCR; OS: overall survival; EMT: epithelial mesenchymal transition; EGFR: epidermal growth factor receptor; HER2: human epidermal growth factor receptor 2; gDNA: genomic DNA; ALU repeats: arthrobacter luteus repeats; CNA: copy number alteration; DDFS: distant disease free survival; DFS: disease free survival; NGS: next generation sequencing; RTq-PCR: real time quantitative-PCR; MSP: methylation specific PCR.

Specific methylation, such as SOX17 promoter methylation in CTCs and ctDNA, can also be utilized as an indicator of early-stage breast cancer [50]. In a study conducted by Chimonidou et al., SOX17 promoter methylation was detected in 86% of primary breast tumors and exhibited a strong correlation between methylation patterns in 114 paired CTC and cfDNA samples, demonstrating that cfDNA largely originates from CTCs or at least the same tumor region from which CTCs have originated. Patients with methylated SOX17 in both cfDNA and CTCs revealed a strong association with breast cancer progression compared to those without methylation. The combined use of cfDNA and CTCs improved the diagnostic accuracy by integrating cellular and genetic information, offering complementary advantages over single biomarker analysis.

### 4.3. Lung Cancer

Lung cancer is one of the leading causes of cancer-related mortality worldwide, with non-small cell lung cancer (NSCLC) accounting for the majority of cases [51,52]. Due to its aggressive progression and the frequent occurrence of late-stage diagnoses, early detection and precise therapeutic monitoring remain critical for enhancing patients’ survival outcomes [53]. Tissue biopsies in lung cancer are often challenging due to the anatomical location of the tumor and patient comorbidities, demonstrating the need for minimally invasive diagnostic approaches. While liquid biopsy techniques provide promising alternatives, single-biomarker approaches face challenges in lung cancer [54,55]. CTC detection is limited by the low abundance of these cells in circulation, particularly in early stages, while ctDNA analysis may not fully capture the phenotypic heterogeneity of tumor cells. The integration of CTC and ctDNA analysis provides complementary evidence about the tumor, enabling a more comprehensive understanding of tumor biology and facilitating treatment stratification for lung cancer patients.

Moon et al. investigated the diagnostic capability of CTCs and ctDNA for detecting primary lung cancer using blood samples obtained from 111 patients, including 99 with primary lung cancer and 12 with benign pulmonary disease (Table 2) [56]. CTCs were isolated using a Cytogen CTC isolation system (size-based filtration), and ctDNA was analyzed using targeted NGS, composed of a 54-gene panel. The diagnostic sensitivity of ctDNA (72.7%) and CTCs (65.7%) was significantly improved when two markers were combined (95.0%), also outperforming conventional tumor markers, such as serum carcinoembryonic antigen (CEA) or CYFRA (cytokeratin fragments). A subgroup analysis confirmed the enhanced sensitivity of the combined CTC/ctDNA assay across all tumor stages and histopathology types. These findings highlight the complementary nature of CTCs and ctDNA, suggesting that their combination can enable the early diagnosis of lung cancer.

Various tumor-specific mutations are investigated from paired CTCs and ctDNA to improve their clinical utility for the diagnosis of lung cancer. Kong et al. conducted a combined analysis of CTCs and ctDNA to explore metastatic heterogeneity and genomic changes in 16 lung and 21 breast cancer patients [57]. CTCs were isolated using size-based enrichment (ClearCell FX1), followed by single-cell isolation using the DropCell platform, while ctDNA was extracted from plasma using commercially available cfDNA extraction kits. The targeted amplicon sequencing of 45 genes for lung cancer and 58 genes for breast cancer revealed that 78% of CTCs from lung cancer patients and 91% of CTCs from breast cancer patients shared at least one mutation with the matched tumor, whereas ctDNA shared mutations with the tumor in 100% of cases. Notably, mutations exclusive to CTCs and ctDNA were identified, reflecting tumor heterogeneity and highlighting their complementary role in disease monitoring. The study also demonstrated that CTCs and ctDNA exhibited a higher degree of concordance with metastatic tumors than primary tumors, emphasizing their potential utility in tracking evolving genomic changes during treatment and progression.

**Figure 3:**
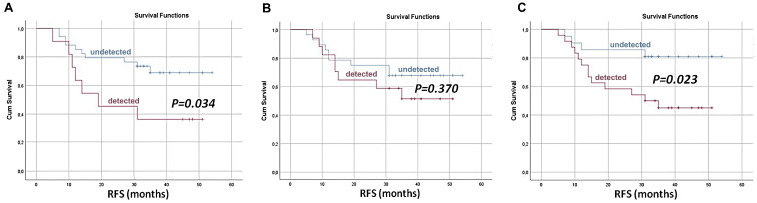
Enhanced prognostic capability through co-analysis of tumor-associated mutations in the following: (**A**) cfDNA, (**B**) CTC-derived DNA, and (**C**) both for predicting recurrence in early-stage NSCLC patients (reproduced under CC BY 4.0 DEED license, Copyright 2023, MDPI) [58].

Likewise, Markou et al. conducted a clinical pilot study to investigate whether preoperative mutational analyses of CTC-derived DNA and cfDNA could serve as complementary markers for predicting relapses in early-stage NSCLC [58]. Blood samples were collected from 49 patients prior to surgery, and hotspot mutations in BRAF, EGFR, KRAS, and PIK3CA genes were analyzed using digital PCR. Mutations were detected in 24.5% of plasma-cfDNA and 38.8% of CTC-derived DNA samples, with PIK3CA being the most frequently mutated gene. A combined analysis increased the detection rates to 53%, highlighting the complementary value of both biomarkers. Patients with detectable mutations in plasma cfDNA exhibited significantly shorter relapse-free survival (RFS) (HR = 2.716, *p* = 0.043). This risk further increased when mutations were identified in either CTC-derived DNA or plasma cfDNA (HR = 3.375, *p* = 0.034), indicating that an integrated liquid biopsy analysis of cfDNA and CTCs improves relapse prediction in early-stage NSCLC (Figure 3).

In another study, Liu et al., analyzed EGFR *mutations* from both CTCs and ctDNA that had been obtained from the blood samples of NSCLC patients [59]. Among 24 patients with EGFR mutations identified in primary tissue, mutations were detected in 45.8% of cases using either CTCs or ctDNA: 7 patients had mutations in ctDNA alone, including key actionable mutations, such as an Exon 19 deletion and L858R; 1 patient had an Exon 19 deletion detected in CTCs alone; and 3 patients demonstrated concordant mutations in both biomarkers. A direct comparison with the tissue biopsy revealed a 54% concordance rate (13 out of 24 patients), with discrepancies likely attributed to the temporal gap, as some tissue biopsies were performed up to 7 years prior to blood collection. This gap indicates the evolving nature of tumor genetics over time, where a liquid biopsy offers a real-time, non-invasive alternative for monitoring molecular changes. Although the overall concordance rate between a tissue and liquid biopsy remains moderate, this study indicates that integrating both biomarkers enhances the molecular profiling of the primary tumor and improves the likelihood of detecting clinically relevant mutations, offering clinically significant precision for therapy selection and monitoring treatment resistance.

### 4.4. Gastrointestinal Cancer

GI cancers, including colorectal cancer (CRC), gastric cancer (GC), and hepatocellular carcinoma (HCC), exhibit significant challenges in oncology due to their high incidence, mortality rates, and associated complexities in diagnosis and management [60,61]. These cancers are often characterized by substantial tumor heterogeneity and metastatic potential, which complicates their diagnosis and management [62]. Liquid biopsy techniques, such as CTC and ctDNA analyses, have emerged as an alternative approach for the non-invasive monitoring of GI cancers [63]. However, single-biomarker approaches also face significant limitations in these cancers. For instance, CTC detection is hindered by low capture rates and phenotypic heterogeneity, while ctDNA analysis may overlook protein-level or phenotypic changes that influence therapeutic resistance. By integrating CTCs and ctDNA, a more comprehensive evaluation of tumor dynamics, progression, and treatment responses can be achieved, addressing these limitations effectively.

CRC is one of the most frequently studied GI cancers using liquid biopsy biomarkers. Salvianti et al. investigated the prognostic value of CTCs and cfDNA in 20 patients with metastatic colorectal cancer (mCRC) harboring KRAS mutations (Table 3) [64]. At the baseline, CTCs were detected in 39% of patients, with CTC positivity significantly associated with a worse PFS (HR = 3.7, *p* = 0.02) and OS (HR = 3.9, *p* = 0.04). cfDNA sequencing revealed mutations in key CRC driver genes, such as KRAS, TP53, APC, and PIK3CA, with TP53 mutations found in 70% of samples. The KRAS mutational status demonstrated 89.5% concordance between cfDNA and tumor tissue, confirming the clinical reliability of the cfDNA analysis for CRC. Longitudinal monitoring revealed that increases in cfDNA levels and the reappearance of CTCs anticipated or overlapped radiological disease progression.

In another study, Takeda et al. detected tumor-associated mutations in DNA obtained from CTCs and cfDNA for the diagnosis of CRC, specifically using NGS and digital PCR [65]. Among 34 untreated patients, 53 mutations were detected in tumor tissues, 47 in cfDNA (including 20 mutations not found in tissues), and 16 in CTC DNA (including 5 unique mutations). Mutations absent in tumor tissues were identified in 35.3% of cases: 26.5% from cfDNA alone and 8.8% from CTC DNA alone, confirming the enhanced sensitivity of combining the two biomarkers. Specifically, in 22 stage IV CRC patients with RAS mutations, digital PCR detected RAS mutations in 63.6% of cfDNA samples and 36.4% of CTC samples. All CTC-positive cases were also positive in cfDNA, and cfDNA generally showed higher variant allele frequencies (VAFs) compared to ctDNA. Importantly, combining cfDNA and CTC analyses improved the overall sensitivity of detecting RAS mutations, as some cases with low cfDNA concentrations could still reveal mutations through CTCs.

Likewise, Kidess-Sigal et al. conducted mutational analysis of CTCs and ctDNA in 15 metastatic CRC patients undergoing liver metastasectomy [66]. Using the label-free Vortex microfluidic platform, CTCs were isolated and processed for the analysis of KRAS, BRAF, and PIK3CA mutations via Sanger sequencing, while ctDNA was analyzed using SCODA enrichment followed by targeted sequencing. CTCs were detected in 80% of blood samples, with an average of 3.4 cells/mL. Mutations were identified in 77.8% of patients, with concordance rates of 78.2% for KRAS, 73.9% for BRAF, and 91.3% for PIK3CA between CTCs and ctDNA. Interestingly, CTCs exhibited mutations not detected in ctDNA in some cases and vice versa, suggesting tumor heterogeneity and the complementary nature of these biomarkers. A longitudinal analysis demonstrated that CTC counts and ctDNA levels paralleled disease progression and treatment responses, often preceding imaging analysis. Altogether, these findings indicate the clinical utility of combining CTC and ctDNA analyses for real-time monitoring and mutation detection in CRC patients.

A co-analysis of CTCs and cfDNA has been conducted in other types of GI cancers, including GC. Yu et al. evaluated the prognostic value of CTCs and cfDNA in 45 patients with advanced gastric cancer (AGC) undergoing neoadjuvant chemotherapy (PSOX regimen) and/or surgery [67]. CTCs were classified into epithelial, mesenchymal, and mixed types using RNA in situ hybridization. The CTC positivity rate was 95.6%, with 51.1% of patients exhibiting mesenchymal CTCs, which were significantly associated with an advanced N-stage (*p* = 0.034) and poor chemotherapy responses. Also, patients with fewer mesenchymal CTCs before treatment were more likely to achieve a partial response (PR) (*p* < 0.05). Meanwhile, a cfDNA analysis revealed that patients achieving a PR had higher baseline cfDNA concentrations, which tended to decrease or remain unchanged after chemotherapy (*p* = 0.119), while higher cfDNA concentrations post-treatment correlated with stable disease (SD) or progressive disease (PD) (*p* = 0.045). Although no direct correlation was found between CTCs and cfDNA, both biomarkers independently predicted the treatment efficacy and prognosis, highlighting their complementary roles in monitoring the treatment responses of AGC.

Similarly, the diagnostic and prognostic capabilities of CTCs and ctDNA have been compared and analyzed for pancreatic cancer [68]. Specifically, CTC counts and the KRAS mutation status in ctDNA were analyzed from 45 pancreatic ductal adenocarcinoma (PDAC) patients, and associations with patients’ pathological features and survival outcomes were extensively explored. CTCs were detected in 20% of patients, predominantly those with metastatic disease, and their presence was associated with a significantly shorter OS (88 days vs. 393 days, *p* = 0.01). KRAS mutations (G12D, G12V, G12R) in cfDNA were detected in 26% of patients using digital PCR, including those with resectable, locally advanced, and metastatic disease. KRAS mutation positivity in cfDNA was also strongly correlated with a poor OS (60 days vs. 772 days, *p* = 0.001). This study also revealed that the cfDNA concentration tended to increase with advanced disease stages, although not statistically significant. Additionally, KRAS mutations were identified in CD45-negative enriched blood samples from patients who were negative for CTCs, indicating that alternate methods for CTC isolation/analysis can further improve the clinical utility of these biomarkers. These findings suggest that combining CTC detection and cfDNA analysis can provide complementary prognostic information and aid in the management of PDAC.

### 4.5. Other Types of Cancer

**Figure 4:**
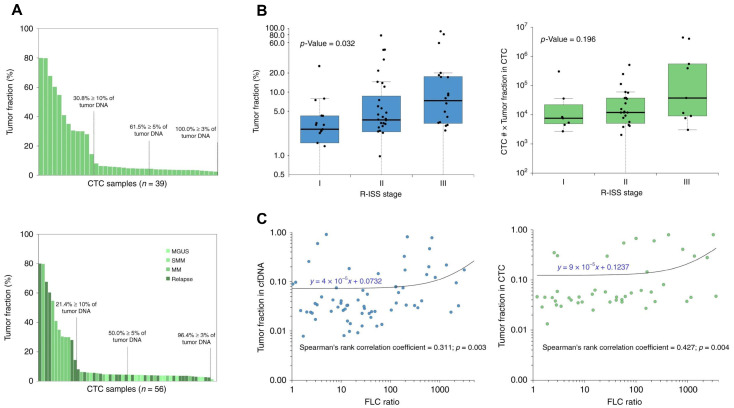
(**A**) ULP-WGS tumor fraction estimates for cfDNA (blue) and CTC samples (green) from multiple myeloma (MM) and relapse patients; (**B**) tumor fractions of cfDNA and CTC samples depending on pathological stage of MM patients; (**C**) tumor fractions of cfDNA and CTC counts depending on the FLC ratio of MM patients (reproduced under CC BY 4.0 DEED license, Copyright 2018, Springer Nature) [69].

The combined analysis of CTCs and cfDNA has been conducted for the diagnosis and prognostic monitoring of other types of cancer, including prostate cancer (PCa), urothelial cancer (UC), and multiple myeloma (MM). For MM specifically, Manier et al. examined the utility of CTCs and cfDNA as biomarkers for estimating the genomic profiles in MM (Table 4) [69]. Among 107 cfDNA and 56 CTC samples analyzed, whole-exome sequencing (WES) demonstrated high concordance with matched bone marrow biopsies for clonal somatic mutations (~99%) and copy number alterations (CNAs; ~81%). CTCs and cfDNA exhibited complementary advantages, with CTCs providing higher tumor fractions in some cases and cfDNA yielding higher fractions in others. The integration of both biomarkers increased the fraction of patients with sufficient tumor DNA for WES analysis, broadening its clinical applicability (Figure 4). Sequential cfDNA monitoring correlated with disease progression and/or therapeutic responses, suggesting its potential for tracking clonal evolution. Importantly, unique mutations were identified in either cfDNA or CTCs that were undetected in tumor biopsies, highlighting the combined analysis as a non-invasive and comprehensive approach for profiling tumor heterogeneity in MM.

The clinical utility of CTCs and ctDNA has been also investigated for PCa. In a study conducted by Schwarzenbach et al., the analysis of blood samples obtained from 81 PCa patients (69 localized, 12 metastatic) revealed that plasma DNA concentrations were significantly higher in metastatic patients (median 562 ng/mL) compared to localized cases (median 186 ng/mL, *p* = 0.03) [70]. Meanwhile, CTCs were detected in 71% of localized PCa and 92% of metastatic PCa patients, with CTC numbers correlating with higher Gleason scores (*p* = 0.04) and advanced tumor stages (*p* < 0.03). Furthermore, microsatellite analysis from cfDNA demonstrated allelic imbalances (AI) in 45% of localized PCa and 58.5% of metastatic PCa cases, with specific markers (D8S137, D9S171, D17S855) exhibiting strong correlations with the presence of CTCs (*p* = 0.03, 0.04, and 0.02, respectively). These findings suggest that both biomarkers can be employed for tracking micrometastatic spread and disease progression in PCa patients.

In another study, dual biomarker analysis was conducted for the diagnosis of UC, including upper tract urothelial cancer (UTUC) and bladder cancer (BC) [71]. Blood samples from 6 patients and urine samples from 10 patients were analyzed using the positive-enrichment platform for the isolation of CTCs, followed by NGS analysis. CTCs were detected in all 6 blood samples, with a median count of 303 CTCs per 10 mL of blood (range 11–676). Mutations were identified in 13/16 (81.3%) of cases, with a median of 18 mutations per patient (range 12–25). Hotspot mutations in genes, such as KIT, PDGFRA, HRAS, MLH1, FGFR3, and TP53, were found in both cfDNA and CTCs, while some mutations (e.g., TP53 and FGFR3) were exclusive to cfDNA. A urinary cfDNA analysis showed a detection rate of 62.5%, with hotspot mutations in PIK3CA, TP53, and MLH1, among others. These findings highlight the potential of a combined CTC and cfDNA analysis to uncover clinically actionable mutations and improve biomarker development for various tumor types.

## 5. Limitations and Prospects

Despite the increasing attention in utilizing CTCs and ctDNA as dual biomarkers in liquid biopsies, several limitations remain (Table 5). While integrating both biomarkers can partially address the limitation of individual biomarkers—such as the cellular phenotypic insights provided by CTCs complementing the high sensitivity of ctDNA—challenges related to their low abundance, variability, and the complexity of co-analysis still hinder their effective application in clinical trials. Furthermore, the standardization and reproducibility of dual-biomarker workflows remain significant barriers.

To address these limitations, integrating additional biomarkers, such as immune cells, serum antigens, exosomes, and microRNAs, has shown promise. Combining CTCs and ctDNA with these emerging biomarkers can enhance the diagnostic accuracy and prognostic capability by providing a more comprehensive picture of the tumor biology. For instance, immune cell profiling and miRNA detection have demonstrated potential to complement CTC and ctDNA analyses [72,73,74]. In another study, the profiling of both CTCs and exosomes has demonstrated enhanced diagnostic capability for diagnosing melanoma [75]. These strategies hold the potential to significantly increase the clinical utility of liquid biopsies in oncology.

Advanced computational platforms, such as machine learning algorithms and artificial intelligence (AI), have emerged as powerful approaches for optimizing the co-analysis of CTCs and ctDNA. These techniques can integrate multi-omic datasets, identify patterns, and improve the sensitivity and specificity of dual-biomarker approaches. For example, the study conducted by Bu et al. highlights the ability of AI-based methods to uncover correlations across biomarkers (e.g., CTC, ctDNA, and exosomes), aiding in more accurate predictions of disease progression and treatment responses [76]. The application of ultra-sensitive biosensors or assays can further enhance the clinical applicability of dual biomarker strategies. These technologies can reduce labor and time consumption while improving the detection limits of CTCs and ctDNA. Advances in microfluidics and nanoparticle-based platforms represent promising avenues for more efficient and scalable liquid biopsy methods [77].

In addition, the simultaneous isolation of both biomarkers can enhance their clinical applicability. Yang et al. developed a system using centrifugal microfluidic technology to directly separate PBMC layers from serum. While the study focused solely on isolating CTCs, the separated serum layer can also be analyzed for cfDNA. This approach has the potential to enable the co-analysis of both biomarkers using a single assay, which efficiently separates serum and PBMC layers. Such a system can minimize sample processing times and helps preserve biomarker integrity [78].

The combined analysis of CTCs and ctDNA offers a minimally invasive approach for comprehensive tumor characterization. Although the current results may not yet be sufficient for immediate clinical implementation, addressing current limitations through biomarker integration, advanced analytics, and technological innovations will pave the way for broader clinical implementation. By leveraging these advancements, liquid biopsy has the potential to transform cancer diagnosis, monitoring, and personalized treatment strategies, ultimately improving patient outcomes.

## Figures and Tables

**Table 2:** Combined analysis of circulating tumor cells and circulating tumor DNA for the diagnosis and molecular profiling of lung cancer.

Type	Samples	CTC		ctDNA		Summary	Ref.
		Isolation	Analysis	Extraction	Analysis		
LC	111 individuals (99 LC, 12 benign)	Cyogen CTC isolation system (Size-based filtration)	IF staining (EpCAM+, CK+, DAPI+, CD45−)	Chemagic cfDNA 5k kit special H24	Targeted NGS for somatic mutations using a 54-gene panel	Combined analysis achieved a sensitivity of 95%, outperforming CTCs (66%), ctDNA (73%), and other serum markers (CEA, CYFRA 21-1 at 66.7%).	[56]
LC, BC	12 LC patients and 12 BC patients	ClearCell FX1 (Size-based Isolation) followed by single-cell isolation using DropCell platform (CD45−negative enrichment)	Targeted amplicon sequencing of gDNA from CTCs using QIAseq DNA Panel for CD45-negative cells with specific morphology	QIAamp Circulating Nucleic Acid Kit	Targeted NGS for somatic mutations using a 45-gene panel for LC and 58 gene panel for BC	Significant mutational heterogeneity observed between the paired CTCs and ctDNA samples. Variants in *TP53*, *PIK3CA*, and *ESR1* identified.	[57]
Early-stage NSCLC	28 SCC, 18 ADC, 3 NOS carcinoma patients	Parsortix System (size-based isolation) followed by single-cell isolation using DropCell platform (CD45−negative enrichment)	Mutational analysis of CTC-derived DNA using ddPCR	Qiagen Circulating Nucleic Acids Kit	Hotspot mutations in *BRAF*, *EGFR*, *KRAS*, and *PIK3CA* genes analyzed using ddPCR	Mutations detected in 53% of patients when both CTC and ctDNA were assessed. Detection of mutations in either biomarkers associated with higher recurrence rates	[58]
NSCLC	24 NSCLC patients and 6 HDs	Vortex microfluidic platform (size-based isolation)	IF staining (CK+, DAPI+, CD45−) EGFR mutations analyzed using EntroGen ctEGFR assay.	QIAamp Circulating Nucleic Acid Kit	*EGFR* mutation profiling using qPCR-based EntroGen ctEGFR assay targeting Exon 19 del, L858R, and T790M mutations.	Combining ctDNA and CTCs provided complementary information for *EGFR* mutation detection.	[58]

CTCs: circulating tumor cells; ctDNA: circulating tumor DNA; cfDNA: cell free DNA; EpCAM: epithelial cell adhesion molecule; CK: cytokeratin; IF staining: immunofluorescence staining; LC: lung cancer; BC: breast cancer; NSCLC: non-small cell lung cancer; SCC: squamous cell carcinoma; ADC: adenocarcinoma; NOS: not otherwise specified; HDs: healthy donors; gDNA: genomic DNA; ddPCR: droplet digital PCR; EGFR: epidermal growth factor receptor; NGS: next generation sequencing; qPCR: quantitative PCR; CEA: carcinoembryonic antigen.

**Table 3:** Combined analysis of circulating tumor cells and circulating tumor DNA for the diagnosis and molecular profiling of gastrointestinal cancer.

Type	Samples	CTC		ctDNA		Summary	Ref.
		Isolation	Analysis	Extraction	Analysis		
mCRC	20 mCRC patients with *KRAS* mutations	CellSearch System (EpCAM-positive selection) followed by single-cell isolation using DEPArray™	Mutational analysis performed via WGA using the Ampli1 WGA kit followed by NGS targeting hotspot regions in 50 oncogenes and tumor suppressor genes	QIAsymphony Circulating DNA Kit.	Targeted NGS for detecting SNVs and indels in 14 CRC-relevant genes using Oncomine Colon cfDNA Assay	CTC positivity at baseline predicted worse OS, while cfDNA enabled reliable detection of KRAS mutations. Increases in cfDNA and CTC counts associated with disease progression.	[64]
CRC	56 CRC patients (34 untreated, 22 stage IV with RAS mutations)	Cynvenio Biosystems LiquidBiopsy Platform (targeting EpCAM, Her2, and Trop2)	IF staining (CK+, CD45−, DAPI+) RAS mutations analyzed using ddPCR NGS for the mutation analysis on 50 oncogenes (Ion AmpliSeq Cancer Hotspot Panel v2)	QIAamp Circulating Nucleic Acid Kit	RAS mutations analyzed using ddPCR NGS for the mutation analysis on 50 oncogenes (Ion AmpliSeq Cancer Hotspot Panel v2)	Combining cfDNA and CTC improved sensitivity for detecting mutations, identifying mutations not found in tumor tissue.	[65]
mCRC	15 mCRC patients undergoing liver metastasectomy (41 blood samples)	Vortex Microfluidic Platform (size-based isolation)	IF staining (CK+, CD45−, DAPI+) Mutation analysis using PCR targeting hotspot mutations in the *KRAS*, *BRAF*, and *PIK3CA*	QIAamp Circulating Nucleic Acid Kit	Mutations analyzed using SCODA mutation enrichment followed by targeted sequencing for *KRAS*, *BRAF*, and *PIK3CA* mutations	Mutations were detected in 77.8% of patients. Concordance rates were 78.2% for *KRAS*, 73.9% for *BRAF*, and 91.3% for *PIK3CA* between CTCs and ctDNA.	[66]
AGC	45 AGC patients undergoing neoadjuvant chemotherapy and surgery	CanPatrol system (nanomembrane filtration)	RNA in situ hybridization to detect epithelial (EpCAM, CK8/18/19), mesenchymal (vimentin, twist), and mixed CTCs.	KminTrak plasma extractor	Quantitatively measured using Qbit fluorescence method.	Mesenchymal CTC levels correlated with advanced N stage and poor chemotherapy response, while higher baseline cfDNA levels predicted better sensitivity to chemotherapy.	[67]
PDAC	45 PDAC patients (31 cfDNA, 35 CTCs)	CellSearch System (EpCAM-positive selection) followed by Immunomagentic depletion of CD45+ cells	IF staining (CK+, CD45−, DAPI+) KRAS mutation analysis Sanger sequencing (G12D, G12R, and G12V)	QIAamp Circulating Nucleic Acid Kit	KRAS mutations (G12D, G12V, G12R) detected via ddPCR	cfDNA was detected across all disease stages, while CTC detection was limited to advanced stages.	[68]

CTCs: circulating tumor cells; ctDNA: circulating tumor DNA; cfDNA: cell free DNA; EpCAM: epithelial cell adhesion molecule; CK: cytokeratin; IF staining: immunofluorescence staining; CRC: colorectal cancer; mCRC: metastatic colorectal cancer; AGC: advanced gastric cancer; PDAC: pancreatic ductal adenocarcinoma; DEP: dielectrophoresis; WGA: whole-genome amplification; NGS: next generation sequencing; PCR: polymerase chain reaction; ddPCR: droplet digital PCR; SNV: single-nucleotide variant.

**Table 4:** Combined analysis of circulating tumor cells and circulating tumor DNA for the diagnosis and molecular profiling of melanoma, urothelial cancer, and pancreatic cancer.

Type	Samples	CTC		ctDNA		Summary	Ref.
		Isolation	Analysis	Extraction	Analysis		
MM	139 MM patients (107 cfDNA, 56 CTC)	Anti-CD138 magnetic bead-based positive selection	Clonal somatic mutations and CNAs detected using ULP-WGS and WES	Qiagen Circulating Nucleic Acids Kit	Clonal somatic mutations and CNAs detected using ULP-WGS and WES	CTCs and cfDNA demonstrated complementary profiles, with some mutations unique to each. Sequential cfDNA monitoring correlated with disease progression and therapeutic responses.	[69]
PCa	81 PCa patients (69 localized, 12 metastatic)	RosetteSep negative enrichment	PSA-EPISPOT assay (PSA-secreting tumor cells)	QIAamp Circulating Nucleic Acid Kit	Microsatellite analysis using PCR	cfDNA levels were significantly higher in metastatic patients compared to localized cases. Allelic imbalances in cfDNA and CTC presence showed significant associations.	[70]
UC	16 UC patients (6 blood samples from UTUC and 10 urine samples from bladder cancer patients)	Cynvenio LiquidBiopsy Blood Collection Kit (Targeting EpCAM, HER2, EGFR, and Trop2)	IF staining (CK+, CD45−, DAPI+) NGS using Ion AmpliSeq Cancer Hotspot Panel v2 (50 cancer-related genes)	MagMAX Cell-Free DNA Isolation Kit	NGS using Ion AmpliSeq Cancer Hotspot Panel v2 (50 cancer-related genes)	Combined NGS analysis of CTCs and cfDNA identified actionable mutations, with cfDNA revealing additional mutations not found in CTCs	[71]

CTCs: circulating tumor cells; ctDNA: circulating tumor DNA; cfDNA: cell free DNA: EpCAM: epithelial cell adhesion molecule; CK: cytokeratin; IF staining: immunofluorescence staining; MM: multiple myeloma; PCa: prostate cancer; UC: urothelial carcinoma; UTUC: upper tract urothelial carcinoma; EGFR: epidermal growth factor receptor; HER2: human epidermal growth factor receptor 2; Trop2: trophoblast cell surface antigen 2; CNAs: copy number alterations; ULP-WGS: ultra-low pass whole-genome sequencing; WES: whole-exome sequencing; PSA: prostate specific antigen; NGS: next-generation sequencing; PCR: polymerase chain reaction.

**Table 5:** Advantages and limitations of utilizing CTCs and ctDNA together for cancer diagnosis and prognosis.

Type	Advantages	Limitations	Ref.
TNBC	Co-analyzing CTCs and ctDNA provides enhanced predictive accuracy for DFS and DDFS outcomes, as demonstrated by the combined presence of these biomarkers after neoadjuvant chemotherapy being strongly associated with an increased risk of recurrence, which cannot be reliably detected by analyzing either biomarker alone.	The inconsistent detection of CTCs and ctDNA in patients has been described, as individual biomarkers often fail to capture all cases of recurrence; for instance, not all patients with disease relapse had detectable ctDNA or CTCs.	[48]
MBC	Co-analyzing CTCs and ctDNA provides a comprehensive view of tumor heterogeneity, as cfDNA captures a broader range of mutations that reflect both individual CTC profiles and additional mutations potentially acquired during disease progression.	The method is constrained by technical challenges, such as the limited detection of low-frequency mutations in individual CTCs due to allelic distortion or dropout during sequencing.	[34]
mCRC	Co-analyzing CTCs and ctDNA enhances prognostic accuracy in mCRC patients, as the presence of both biomarkers correlates with a poorer PFS and OS, providing a more comprehensive assessment of patient prognosis than either biomarker alone	The detection rates of CTCs and cfDNA can vary among patients, and the absence of one biomarker does not necessarily predict a better prognosis.	[64]
MM	The presence of both biomarkers is associated with a poorer PFS and OS, suggesting that their combined detection can enhance the accuracy of patient prognosis compared to evaluating each biomarker individually.	The approach is limited by variable tumor fractions in CTCs and cfDNA across patients, which can impact detectability.	[69]
PCa	cfDNA provides a broader view of tumor-specific genomic aberrations, such as allelic imbalances, while CTCs offer direct evidence of tumor cell dissemination, together enabling more accurate assessment of tumor progression and metastatic potential.	cfDNA not always correlating with CTC presence due to differences in tumor shedding.	[70]

TNBC: triple-negative breast cancer; MBC: metastatic breast cancer; mCRC: metastatic breast cancer; MM: multiple myeloma; PCa: pancreatic cancer; CTC: circulating tumor cells; ctDNA: circulating tumor DNA; cfDNA: cell free DNA: DFS: disease-free survival; DDFS: distance disease-free survival; OS: overall survival.

## Data Availability

No new data were created in this study. Data sharing is thus not applicable to this article.
